# Practical Notes on Diagnosis and Treatment

**Published:** 1910-01-22

**Authors:** 


### Practical Notes on Diagnosis and Treatment.

### Dietaries.

We should never advise what a patient should eat until we have inquired what he does eat.—*Trousseau.*

### Migraine.

An attack of migraine is often accompanied by marked coldness of the feet, and measures directed to restoring their warmth will also give valuable aid in relieving the headache.

### Insomnia.

A combination of hypnotics is often more successful than any of them singly. A useful prescription is 5 or 10 minims of liquor opii, with 5 grains of chloral and 10 to 30 grains of potassium bromide. *Sir T. Lauder Brunton.*

### Gastralgia.

For a patient who has gastralgic pains, judicious boldness in feeding is often beneficial. Never assume a patient is dyspeptic because he has pains in his stomach. These patients often get worse and worse upon a restricted diet, and the restrictions have been increased improperly.—*Sir James Sawyer.*

### Starvation in Aneurysm.

It is difficult to see how the starving of people is going to promote coagulation. By all means you should control the amount of fluid, but not to such an extent as to make the patient uncomfortable. Keep the fluid down to a pint, but give a generous amount of solids; this I consider is physiological.—*Dr. Goodhart.*

### Cerumen and Hydrogen Peroxide.

When it is desired to remove immediately, by syringing, wax in the ear, drop a little $H_2O_2$ solution into the meatus. This will effectually loosen the wax, and the operation may be completed in one attendance.

### Turpentine as a Hæmostatic.

In hæmatemesis from a gastric ulcer turpentine has been given successfully to control the bleeding. It may be given beaten up with the yolk of an egg or the dose may be emulsified with mucilage. In bleeding from the socket of a tooth, it may be applied locally.

### Epulis.

Roughly two-thirds of epulides are sarcomatous in nature. To avoid recurrence the initial operation should be radical, including removal of part of the alveolar process of the jaw. Lack of care of the mouth and teeth is a very potent factor in predisposing to the growth of epulides; hence the importance of having the teeth periodically overhauled.

### Lead Poisoning.

One must not expeect to find a "blue line" unless the gums are somewhat separated from the teeth by a space in which may lodge decomposing albuminous material, capable of yielding sulphur. With perfect gums you must seek for confirmation in the possible causes—*e.g.* analysis of the drinking water; and the urine should be examined after potassium iodide has been administered for a few days.—*Sir Wm. Gowers.*

